# Supplementary material for: Long-Term Performance of Nanomodified Coated Concrete Structures under Hostile Marine Climate Conditions
Source: Nanomaterials (Basel). 2021 Mar 29;11(4):869. doi: 10.3390/nano11040869 (PMC8066161; doi:10.3390/nano11040869)
Supplement: Supplementary file 1 [file nanomaterials-11-00869-s001.pdf]

nanomaterials

Supplementary Information

Long-Term Performance of Nanomodified Coated Concrete Structures under Hostile Marine Climate Conditions

Adrián Esteban-Arranz [1], Ana Raquel de la Osa [1], Wendy Eunice García-Lorefice [1], Javier Sacristan [2,†], Luz Sánchez-Silva [1*]

[1] Departament of Chemical Engineering, University of Castilla-La Mancha, Avda. Camilo José Cela 12, 13071 Ciudad Real, Spain.; Adrian.Esteban@uclm.es; AnaRaquel.Osa@uclm.es; Wendy.Garcia@uclm.es
[2] ACCIONA Technological Centre, Alcobendas, 28108, Madrid, Spain.
* Correspondence: MariaLuz.Sanchez@uclm.es; +34 926 29 53 00 ext: 6307.
† Present email address: sacristan.javier@es.sika.com

Supplementary Information Tables

Table S1. Main components, description, and mixing ratio of the different resins.

Table S2. Density results, size, surface area values and average pore radius of the different nanoparticles.

Supplementary Information Figures

Figure S1. Coating methodology of concrete: spatula printing; paintbrush impregnation; paintroller impregnation and dip coating.

Figure S2. HRSEM micrographs (a) and contact angle measurements (b) of the selected nanoparticles.

Figure S3. ATR-FTIR spectra of the different nanoparticles.

Figure S4. Optimization of the NPs incorporation into the Part B (hardener) of the coating.

Figure S5. Visual appearance of the concretes coated with the nanomodified epoxy resins.

Figure S6. Contact angle results of the different coatings before and after the weathering test.

Figure S7. Optical loop results of the different coatings before and after the weathering test.

Figure S8. ATR-FTIR spectra of the S10-1 production (a) and the effect of nanoparticles incorporation on the curing reaction of the NPs-S10-1 before the weathering test (b).

Figure S9. TGA profiles of the different NPs-S10-1 composites under nitrogen (a) and air (b) atmospheres before and after the weathering test.

Supplementary Information Tables

Table S1

	Principal Component	Description	Mixing ratio (wt.%)
SE	Bisphenol-A diglycidyl ether resin (60%)	Part A-resin	1 A: 2.4 B: 14 C
	Proprietary mixture of primary amines (100%)	Part B-hardener	
	Cement based	Part C-filler	
S	Bisphenol-A epichlorohydrin (75%)	Part A-resin	3 A: 1 B
	Amine-based components (45%)	Part B-hardener	
	Bisphenol-A diglycidyl ether resin (75%)	Part A-resin	3 A: 1 B
	Epoxy Amine Adduct (40%)/Benzyl alcohol (30%)	Part B-hardener	
M	Calcium dihydroxide (50%)/Ethyleneglycol (50%)	Part A-resin	1.5 A: 3.5 B
	Polymethylene polyphenyl polyisocyanate (90%)	Part B-hardener	
	Calcium dihydroxide (50%)/Ethyleneglycol (30%)	Part A-resin	2.2 A: 2.8 B
	Polymethylene polyphenyl polyisocyanate (80%)	Part B-hardener	

Table S2

	Density ($g \cdot cm^{-3}$)	Size (nm)	S_{BET} ($m^2 \cdot g^{-1}$)	Average pore radius (nm)
AC	1.1±0.0006	101.1	954.8	1.5
SMNC	1.7±0.0002	< 20 μm	11.8	11.9
SiO_2	2.7±0.0003	5-15	532.3	1.7
ZnO	6.1±0.0002	91.8	5.4	9.0

Supplementary Information Figures

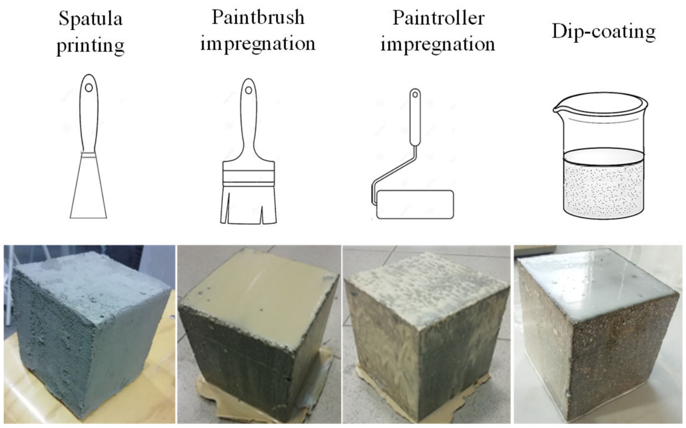

Figure S1.

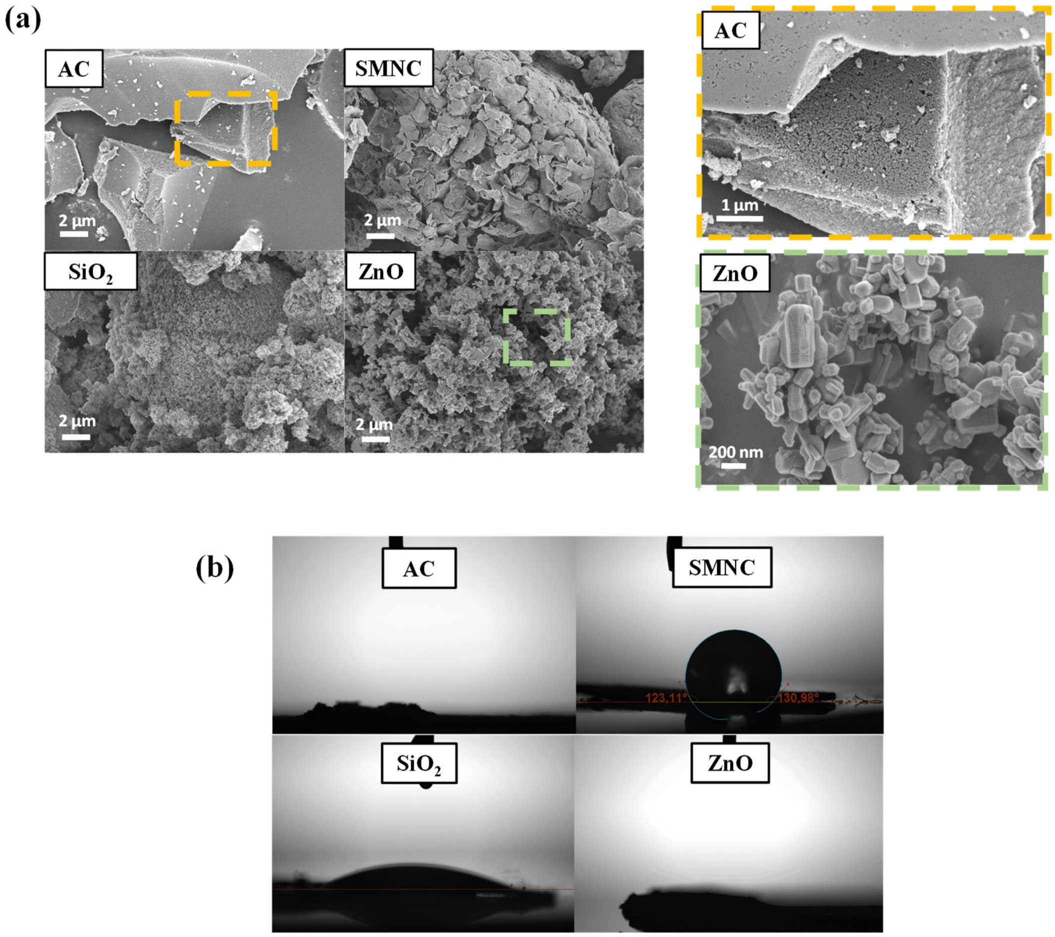

Figure S2.

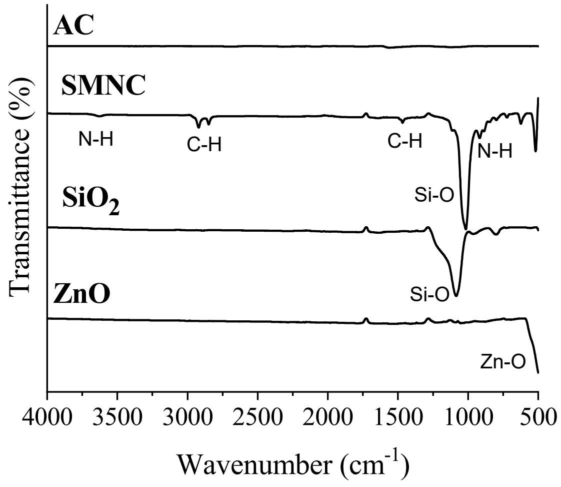

Figure S3.

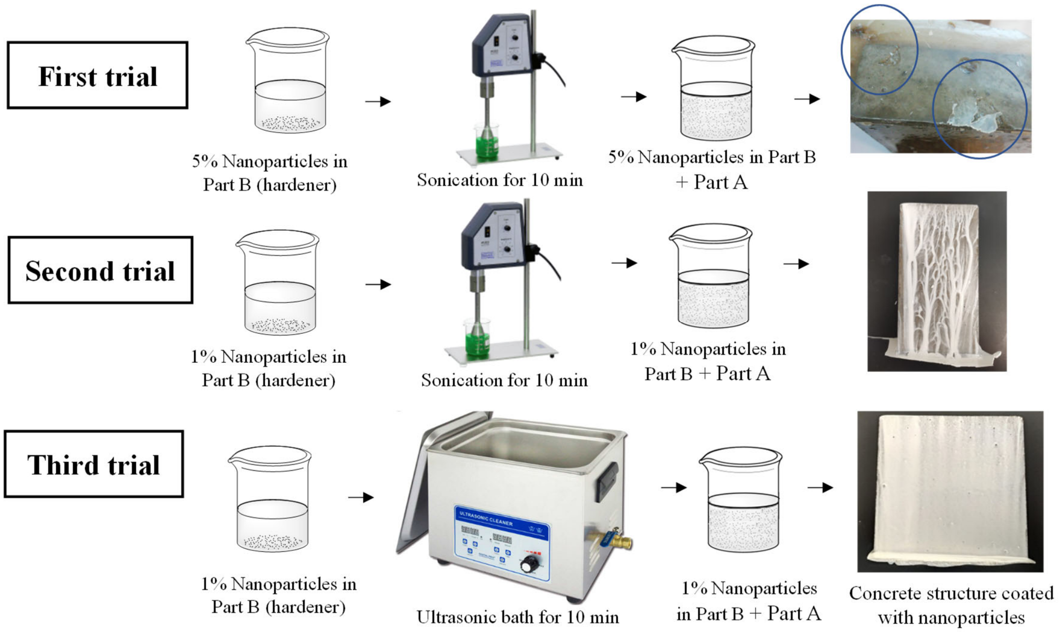

Figure S4.

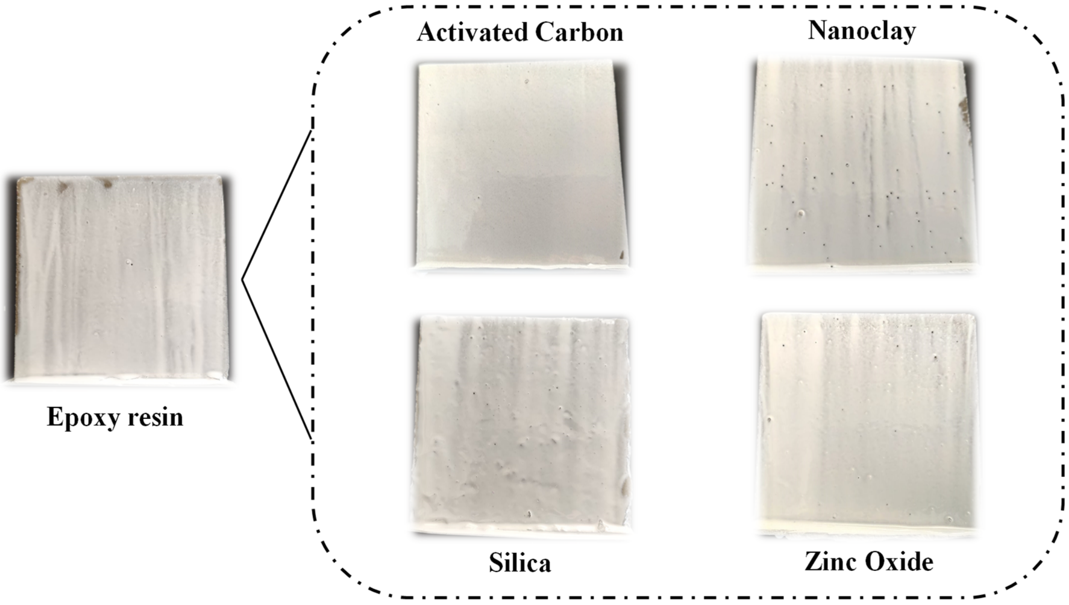

Figure S5.

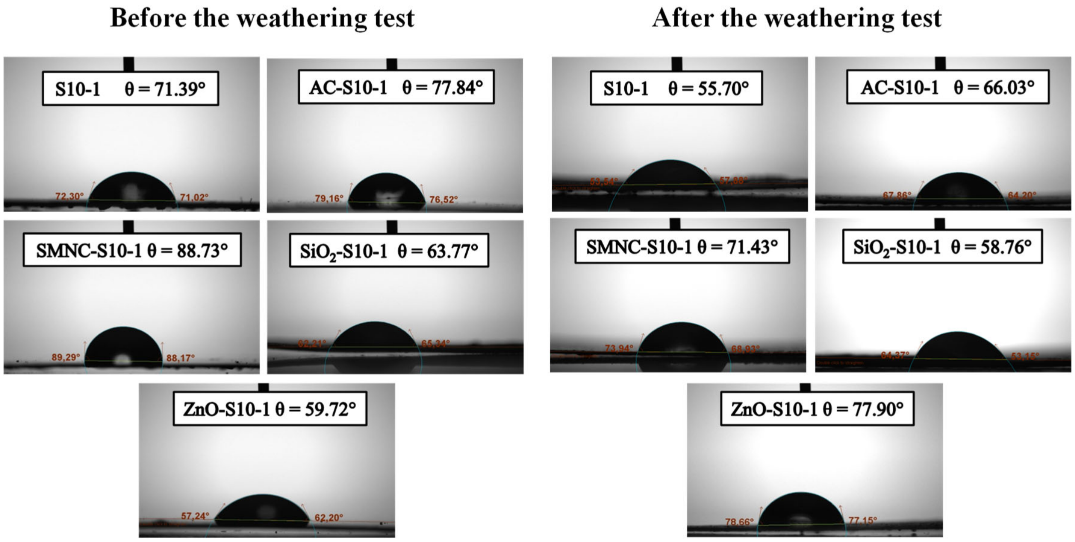

Figure S6.

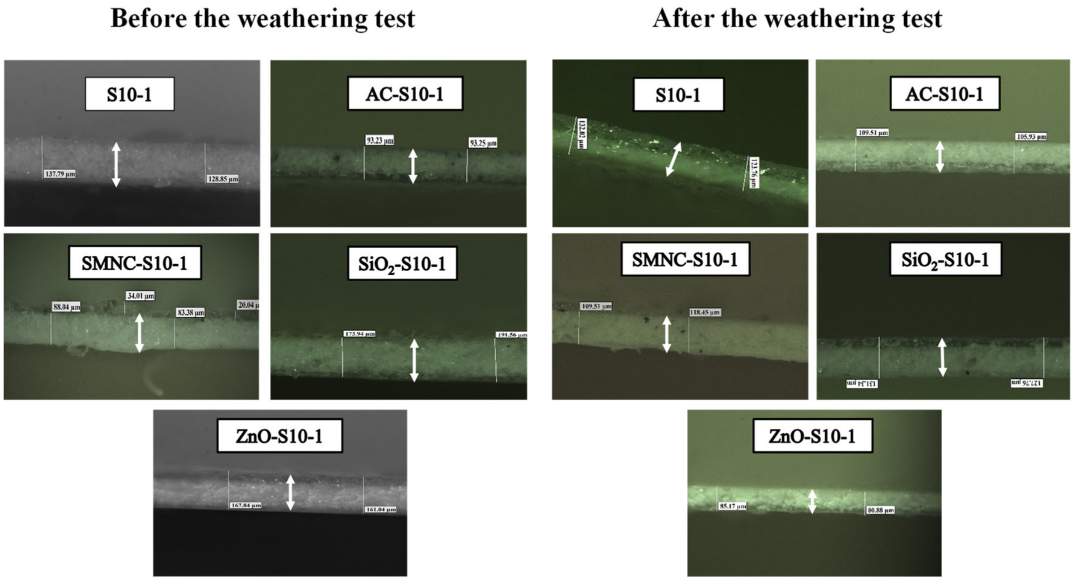

Figure S7.

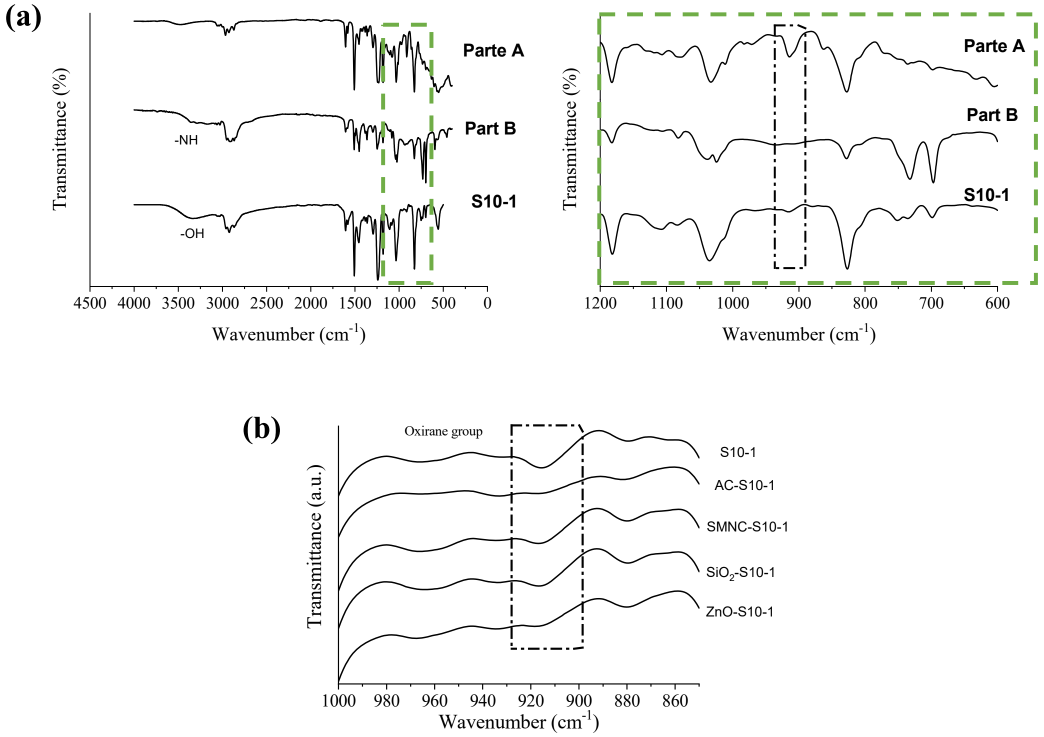
(a)
Transmittance (%)
Parte A
Part B
S10-1
-NH
-OH
4500 4000 3500 3000 2500 2000 1500 1000 500 0
Wavenumber (cm-1)
Transmittance (%)
Parte A
Part B
S10-1
1200 1100 1000 900 800 700 600
Wavenumber (cm-1)
(b)
Oxirane group
Transmittance (a.u.)
S10-1
AC-S10-1
SMNC-S10-1
SiO2-S10-1
ZnO-S10-1
1000 980 960 940 920 900 880 860
Wavenumber (cm-1)

Figure S8.

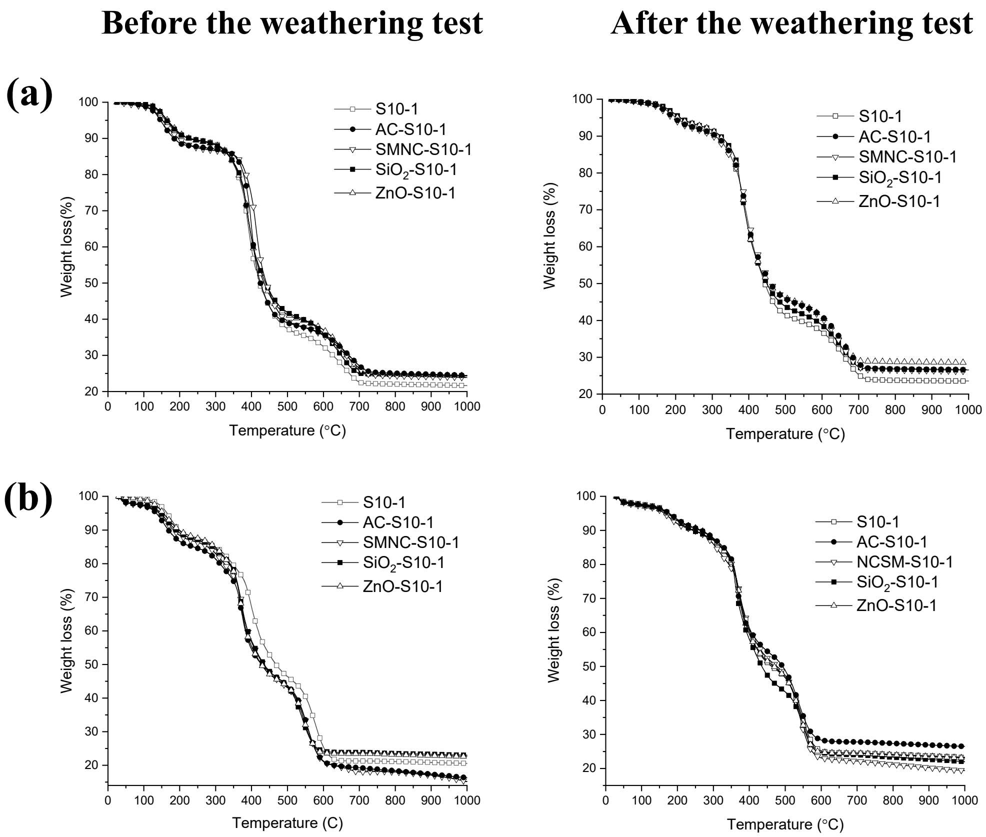
Before the weathering test
After the weathering test
(a)
(b)
S10-1
AC-S10-1
SMNC-S10-1
SiO_2-S10-1
ZnO-S10-1
NCSM-S10-1
Weight loss(%)
Weight loss (%)
Temperature (°C)
Temperature (C)

Figure S9.
